# Cost assessment of health interventions and diseases

**DOI:** 10.1136/rmdopen-2020-001287

**Published:** 2020-11-03

**Authors:** Bruno Fautrel, Annelies Boonen, Maarten de Wit, Sabine Grimm, Manuela Joore, Francis Guillemin

**Affiliations:** 1 Sorbonne Université – Assistance Publique Hôpitaux de Paris, Pitie Salpetriere University Hospital, Rheumatology Department, Paris, France; 2 Institut Pierre Louis d’Epidémiologie et Santé publique, INSERM UMR S1136, Team PEPITES, Paris, France; 3 Department of Internal Medicine, Division of Rheumatology, Maastricht University Medical Center, and the CAPHRI Research Institute Maastricht University, Maastricht, Netherlands; 4 Patient Research Partner, EULAR, Zaltbommel, Netherlands; 5 Department of Clinical Epidemiology and Medical Technology Assessment (KEMTA), Care and Public Health Research Institute (CAPHRI), Faculty of Health, Medicine and Life Sciences (FHML), Maastricht University Medical Centre, Maastricht, The Netherlands; 6 EA 4360 APEMAC, School of Public Health, Nancy, France

**Keywords:** Economics, Epidemiology, Health services research

## Abstract

Health resource use and identification of related costs are two essential steps in health economics assessment. The elicited costs will be balanced with health outcome improvement and enable the comparison of different diagnostic procedures or therapeutic strategies from a health economic point of view. The cost typology can be disentangled in three main components, that is, direct cost related to health resource use, indirect costs related to productivity loss and sometimes intangible costs (costs related to pain and suffering). These costs can be elicited from different perspectives depending on the general aim of the assessment: payer, societal perspective or patient perspective. Practically, the first step corresponds to the quantification of health resource use, that is, number of consultations, biological or imaging workups, hospitalisation, dispensed medication units or days on sick leave. It can be done by specific self-questionnaires or by access to insurance health databases. The second step is then to value each health resource use item, based on available public databases—either produced by insurance entities or statistics institute—providing the unit costs for each item. Importantly, substantial variability does exist in the costing exercise, requiring accepting a certain uncertainty around cost estimates. This can be taken into account by sensitivity analyses, which capture in what extent measurement error can impact cost assessment, depending on different hypotheses or assumptions. One essential element of health economic assessment is the identification of costs incurred by or associated with a specific health condition for a study on the economic burden of a disease—cost-of-illness study—or with a given diagnostic or therapeutic intervention in the context of health technology assessment in which these costs are compared with the alternative reference strategy—cost-effectiveness study.

## COST TYPOLOGY

**Figure 1:**
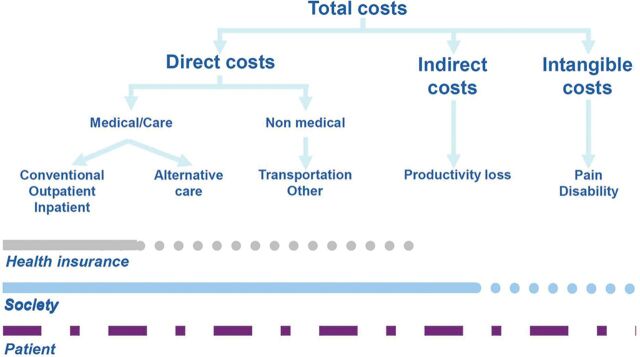
Cost typology.

Health-related costs have been disentangled in three main components^1–5^ (figure 1).

### Direct costs

Direct costs correspond to the costs for caring the patient. They can be disentangled in two subgroups: direct medical costs and direct non-medical costs.

### Direct medical costs

They include many different cost shares, that is,

- visits to the physicians, either general practitioners or specialists;

- visits to other healthcare professionals, such as physiotherapists, occupation therapists, psychologists, nurses, social workers;

- biological, imaging or pathological workups either for disease diagnosis and disease activity assessment, as well as for drug monitoring;

- drug treatments;

- non-drug interventions, that is, surgery or any percutaneous intervention, or any specific intervention related to the disease (rehabilitation, mindfulness meditation, self-help groups, etc);

- inpatient care, that is, hospitalisations whatever the reason.

Importantly, it should be clearly pointed out which costs will be taken into account: all health resource use whatever the reason at its origin, costs directly related to the disease of interest, costs related to comorbidities related to the disease of interest or its treatment.

### Direct non-medical costs

It is also possible to include here additional health interventions such as alternative and complementary medicine, as well as transportation costs when they are covered but the health insurance. These heath resource use items could be justified depending on the objective of the economic assessment: for example, to make a decision on the coverage of complementary and alternative medicines in the basket of a health insurance to include or not alternative therapies; or to compare of hospital-based intravenous therapies (requiring transportation to the hospital) to subcutaneous self-administered therapies.

### Indirect costs

Indirect costs correspond to costs related to productivity loss. The definition of productivity in health economics is definitely not limited to the impact of a health condition on paid activities. A person is ‘productive’ from the societal perspective if this person is useful to society, either due to her/his work and the production of goods related to it, or due to its involvement in non-paid activities such as volunteer work, involvement in non-for-profit organisation or even household or familial duties.

Practically, productivity loss is often focused on absenteeism, and thus limited to the economic impact of days off work (missed work days), either due to sick leave or disability status.^6–9^ Absenteeism is clearly the most obvious and consensual expression of health-related productivity loss, although the attribution of sick leave to a specific health problem may be quite challenging.

**Figure 2:**
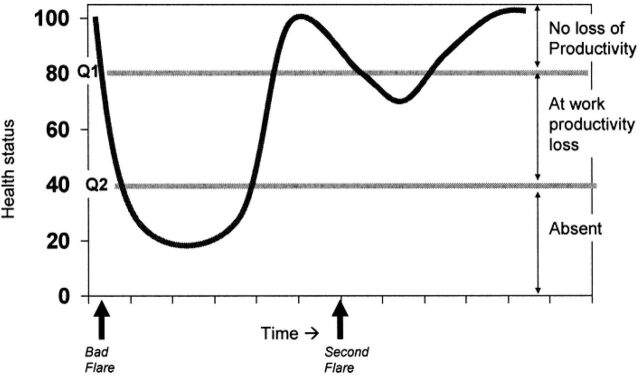
Concepts behind worker productivity: absenteeism and presenteeism.^7^

In addition, the notion of presenteeism has been proposed to quantify and valorise the impact of a disease on patient productivity while still at work on the market place (figure 2). Actually, numerous pieces of evidence identified that people living with a health problem are able to work although they are limited by their disease: it can result in the need of additional work hour to complete the same task or in a reduced production compared to a healthy worker.^7^ Presenteeism raised significant interest for research in health economics but no robust and consensual standards have been defined to date to be used in economic assessments.

### Intangible costs

Intangible costs are a more conceptual cost compound trying to input a cost on pain, suffering and globally on the fact that a person is not in perfect health. Intangible costs are used by specific insurance when they have to compensate a person for an adverse life event (a car accident, eg) but less rarely in health technology assessment and health economics.

## COST ELICITATION

### Perspective

The perspective in which the economic assessment is conducted is central and will determine the typology of costs that have to be included in the analysis. Three main perspectives are used in the literature.

#### Payer

This perspective will limit cost elicitation to those covered and reimbursed by the payer. They can be limited to direct costs only in some healthcare system or may also include indirect costs when the payer is also in charge of daily allowance or disability pension in case of short- or long-term sick leave.

#### Society

The societal perspective aims to use a more global approach including all the aspects that are important to society and its functioning. Here, both direct and indirect costs are taken into account.

#### Patient

In the patient perspective, more attention is dedicated to disease impact on patients and their relatives to manage the disease and its consequences. It can be either co-payments (when the service or health good is only partially covered by the health insurance) or out-of-pocket expenditures (thus not covered by any health insurance); it can be directly related to the care or deal with expenditures requested to adapt to the disease (living arrangement or home adaptation).

Whatever the perspective, cost elicitation requires two steps:

1. The collection of health resource use data, that is, the consumption of health goods by the patients;
2. The valuation of the identified health resource use, by inputing monetary values to each consumed health item.

### Identification of health resource use

The data to be collected for health resource use characterisation have been standardised in the field of rheumatology 20 years ago in the context of an OMERACT initiative.^2 3^ It has been summarised in a reference case for economic assessment (table 1).

**Table 1:** Cost matrix, usable for economic evaluations

Direct costs *Cost of care*	Outpatient costs	- Visits to physicians (specialists and other) - Outpatient surgery - Emergency room visits - Non-physician service utilisation (physiotherapist, occupational therapist, social worker, psychologist, etc) - Medication - Diagnostic or therapeutic procedures and tests - Devices and aids
	Inpatient costs	- Acute hospital facilities (without surgery) - Acute hospital facilities (surgery) - Non-acute hospital facilities (rehabilitation, nursing homes)
	Other disease-related costs (direct)	- Transportation - Home healthcare services - Home remodelling - Medical equipment (non-prescription) - Non-medical practitioner, alternative therapy - Patient time
Indirect costs *Productivity loss*	- Loss of productivity in employed patients (sick leave, work disability) - Opportunity costs (loss of productivity due to time spent by nursing family members, disabilities leading to impaired housekeeping or activities of daily life) - Low wages	

Adapted from Merkesdal *et al.* ^2^

### Data for direct costs

Two principal sources of information can be used: administrative databases of health organisations or information reported by patients themselves through standardised self-questionnaires.

In some countries, there is a large access to hospital, healthcare system or health insurance databases. When possible, it provides all the medical visits, biological or imaging workups and medication deliverance. The quantity of data is often substantial but their quality is of great value—with only minimal missing or incomplete data—and it enables robust estimates of health resource use.

When the access to such data is not feasible, heath resource use is based on self-questionnaires, filled in by patients themselves. These questionnaires explore different items of the cost matrix^2^ and have to be regularly administered to the patients, ranging from every day (patient diary) or monthly—with low memory bias, but high risk of missing data—or biannually or annually—with less missing data, but higher memory bias. Some others have proposed to explore a 1-month period every semester or year, with subsequent extrapolation to a 1-year timeframe, in order to minimise both memory bias and respondent workload; this has never been fully validated to date. Another limitation deals with a certain level of inaccuracy in the responses provided by the respondents, such as the mention of a ‘blood test’ without the specific tests requested (which make the cost of a blood test) or of an imaging workup without information on the site explored.

There is no fully consensual and validated questionnaire to date, but several studies have used a questionnaire derived from the Stanford Health Assessment Questionnaire (Annex).^10–13^ They clearly raise issues with regards to the quality of the collected information and have limitations: memorisation bias, some inaccuracy (eg, notion of imaging without the body site explored or notion of blood tests without the exact test description), lack of precision in the main reason for hospitalisation (only text information without exact ICD code available from the patients), errors in the exact formulation or dosage of a medication, etc.

10.1136/rmdopen-2020-001287.supp1 Supplementary data

### Data required for indirect costs

The same two options are possible.

The access to health insurance databases provides accurate information on the number of days off work in relation to a medical problem, either due to sick leave or disability status (table 2). It is thus a relevant source of data for absenteeism.

**Table 2:** Productivity loss quantification for economic analyses^7^

Perspective	Outcome state	Cost indicator
Component		
Absenteeism	Number of days/hours off work	Cost of time away from job
Presenteeism (at-work productivity loss)	Difficulties at work	Worker productivity loss, expressed in hours and translated to money unit

Alternatively, specific questionnaires have been developed to assess the productivity loss associated with a specific medical. Some questionnaires are focused on absenteeism, that is, on days off work due to short-term sick leave or long-term disability status.^7^ They are rather straightforward and are mainly limited by potential memory biases when the questionnaire addresses long period of time (as for health consumption data). The attribution of a sick leave to a specific health problem can be pointed out by the patient herself or himself, but it always remains questionable for health economists.^6–9 14^

Other questionnaires are dedicated to presenteeism (or a combination of absenteeism and presenteeism). They often express the productivity loss while still at work as a percentage of productivity compared to productivity without the disease.^15–17^ This can then be converted in lost hours or additional hours to perform to achieve the same task (table 2). However, the concept of presenteeism as well as the methodological issues around its assessment make that presenteeism is rarely included in health technology or economic assessments.

Beyond these points, it is important to keep in mind that it is highly difficult to fully capture the exact impact of a disease in terms of productivity loss. The relation between health and productivity is quite complex and largely depends on contextual factors that are difficult to capture.^9 17–19^ Additionally, the evolution of a given person productivity (and thus the resulting productivity loss) in the absence of a given chronic illness is almost impossible to forecast on a long-term basis, once the disease has started.

### Valuation

This step aims to input costs on health resource use and productivity losses incurred by the patients. This task can be simple and straightforward when data are extracted from health insurance databases in which data are always associated with reimbursement data, that is, costs covered by the insurance. In other cases, the task is more time consuming since there is a need to input cost values for every item, that is, consultations, biological or imaging workups, hospitalisation, medication or money compensation for sick leave.

### Direct costs

The cost imputation requires identifying the corresponding fees or tariffs, which can be facilitated by cost tables or matrices. It has to be mentioned that there are frequently several possible costs available for one given health resource item.

These costs can be disentangled depending on the payer: costs covered by universal public health insurance, costs covered by additional non-mandatory insurance and costs remaining to be paid by the patients (out-of-pocket costs).

They are frequently published in open access by the health authorities or insurances at a province or country level, and the existing sources of information can be found on the website of the International Society for Pharmacoeconomics and Outcomes Research (https://tools.ispor.org/htaroadmaps/).

### Indirect costs

The costs associated to days of work can be derived from the patient wage if this information is known or from the average daily wage of the country population; this information is often publicly available on the National Statistics websites. The valuation process is more complex for non-paid activities (domestic tasks, volunteering, etc): the majority of studies uses ‘replacement’ costs, that is, the average wage of a household employee who could perform the same tasks in replacement of the sick household member. Alternatively, some authors proposed to use ‘opportunity’ costs which are increased to take into account the autonomy loss and the difficulty to accept not to be able to perform these activities by oneself.

A specific attention should be dedicated to indirect costs for which two different methods have been proposed by health economists (table 3), resulting in extremely different indirect cost estimates.

**Table 3:** Differences in the two methods used for productivity loss valuation

	Paid activities	Non-paid activities	Limitations
Friction costs	3 months off (replaced after by an unemployed worker)	Non-valorised	Systematic underestimation of specific cost components: costs related to long-term disability or household/non-paid activities, or to geriatric diseases
Human capital	Salary or equivalent	Non-valorised	Systematic underestimation of specific cost components: costs related to household/non-paid activities, or to geriatric diseases
		Replacement costs	Systematic underestimation of costs of diseases affecting predominantly women
		Opportunity costs	Systematic underestimation of costs of diseases affecting predominantly women

In the Friction Cost method (FC), productivity loss is considered only for a maximum period of 3 months: since unemployment is common and substantial in all countries, patients with a sick leave longer than 3 months can be replaced by an unemployed worker and are thus not considered any longer. The first 3-month period during which the sick leave is valued is called the friction period. This method is often preferred when the economic assessment is done in a health insurance (payor) perspective. This results in a substantial impact—underestimation—on indirect cost estimates, especially for chronic and disabling diseases.

In the Human Capital method (HC), the productivity loss is not limited in terms of duration and indirect costs include all productivity costs, that is, sick leave, disability pensions, early retirement (etc), whatever their duration. This method is preferred when the assessment is conducted from a societal or human perspective. Logically, the HC estimates are often dramatically superior to the FC ones, up to 15-fold in a paper directly considering the difference between the two methods.^20^

### Uncertainty around cost estimates

Whatever the method and the data sources used to elicit costs, estimates are subjects to substantial variability and only one figure cannot translate the economic impact of a given health condition and enable comparison between diseases or therapeutic options. Thus, all cost elicitations need to highlight this uncertainty. An option is to provide an average estimate in association with a ‘best-case estimate’ in which all health resource or productivity items is valued with the lowest possible (and realistic) value (best-case scenario) and a ‘worst-case estimate’ in which all health resource or productivity items are valued with the highest possible (and realistic) value (worst-case scenario). An alternate option is to calculate CI around the cost estimates: since cost distribution is never normal, bootstrap methods are to be used.

## CONCLUSION

Cost elicitation in health economic assessment is always a challenging and time-consuming task. The use of consensual methodological standards is the best strategy to obtain robust and credible cost estimates. The elicited cost estimates can then be used to describe the economic impact of either a disease as a whole, or a specific diagnostic or therapeutic strategy in a given medical context. They can also be used to compare them with another disease or another reference strategy: in this context, cost assessments are one of the two pillars of health technology assessment.
