# Supplementary material for: Cost assessment of health interventions and diseases
Source: RMD Open. 2020 Nov 3;6(3):e001287. doi: 10.1136/rmdopen-2020-001287 (PMC7856133; doi:10.1136/rmdopen-2020-001287)
Supplement: Supplementary data [file rmdopen-2020-001287supp001.pdf]

Annex: Example of a self-questionnaire for health resource use elicitation (10,12)

**1- In the PAST MONTH, have you made any visits to any of the following doctors or health professionals?**<br>☐ Yes ☐ No<br>Please complete the following:	
**TYPE of Doctors or Health Professional**	**Number of visits in the past month**
**A) Rheumatologist**	
**B) Internal Medicine Specialist**	
**C) Family doctor**	
**D) Orthopedic Surgeon**	
**E) Podiatrist (foot doctor)**	
**F) Other doctors (e.g. dermatologist, …)**	
**G) Physical Therapist**	
**H) Occupational therapist**	
**I) Chiropractor**	
**J) Other health workers (social worker or others - give type)**	

**2. In the PAST MONTH, have you had any tests or diagnostic procedures performed?**<br>☐ YES ☐ NO<br>If "YES", please complete the following:		
**Test**	**N tests in the past month**	**PART OF BODY**
**A) X-rays (e.g. head, chest, abdomen, limbs)**		
**B) CT-scan**		
**C) MRI scan**		
**D) Blood Tests (N times blood was drawn)**		
**E) Urine tests**		
**F) Gastroscopy (Endoscopy)**		

**G) Colonoscopy**		
**H) Other tests**<br>**(e.g. mammogram, ultrasound, breathing test, joint scan, bone density scan)**<br>**– please specify type of test**<br>**__________________________**		

3. In the PAST MONTH, have you had any hospitalization?

Include all hospitalizations, whether or not they were related to your disease.

☐ YES ☐ NO

If "YES", please list:

Reason	Location Name & City	Date (Month, Year)	N days	ER

4. FILL IN ONLY IF YOU ARE CURRENTLY WORKING FULL OR PART-TIME FOR PAY)

In the PAST MONTH have you missed work because of any medical condition?

In addition to days you spent at home because of illness, include all days you spent as an inpatient in hospital or at a rehabilitation facility, if this applies to you.

___YES ___NO

If "YES",

a) How many days? ______

b) How many, if any, were because of your disease? ______
